# Identification of Posttranslational Modifications in Peroxisome Proliferator-Activated Receptor *γ* Using Mass Spectrometry

**DOI:** 10.1155/2014/468925

**Published:** 2014-06-25

**Authors:** Shogo Katsura, Tomoko Okumura, Ryo Ito, Akira Sugawara, Atsushi Yokoyama

**Affiliations:** ^1^Institute of Molecular and Cellular Biosciences, University of Tokyo, 1-1-1 Yayoi, Bunkyo-ku, Tokyo 113-0032, Japan; ^2^Department of Molecular Endocrinology, Tohoku University Graduate School of Medicine, 2-1 Seiryo-machi, Aoba-ku, Sendai 980-8575, Japan

## Abstract

Posttranslational modification (PTM) of proteins is critical for various cellular processes. However, there are few studies examining PTMs in specific proteins using unbiased approaches. Here we report the attempt to identify the PTMs in peroxisome proliferator-activated receptor *γ* (PPAR*γ*) proteins using our previously established PTM analysis system. In this study, we identified several PTMs in exogenously expressed PPAR*γ*2 proteins from 293T cells as well as endogenous PPAR*γ*1 proteins from a Caco-2 colon cancer cell line. The identified PTMs include phosphorylation of serine 112 and serine 81 in PPAR*γ*2 and PPAR*γ*1, respectively, both of which are well-known mitogen-activated protein kinase- (MAP kinase-) mediated PTMs in PPAR*γ* proteins, thus confirming our experimental approach. Furthermore, previously unknown PTMs were also identified, demonstrating that this method can be applied to find previously unidentified PTMs in PPAR*γ* proteins and other proteins including nuclear receptors.

## 1. Introduction

Peroxisome proliferator-activated receptor *γ* (PPAR*γ* and NR1C3) is a ligand-dependent nuclear receptor, which was initially established as the dominant regulator of adipocyte differentiation [1, 2]. PPAR*γ* is activated by natural ligands, such as polyunsaturated fatty acids and eicosanoids, plays a dominant role in adipose cell differentiation, modulates metabolism and inflammation in immune cells, and has strong antigrowth properties. Although PPAR*γ* levels are the highest in adipose cells, substantial levels are also found in certain other cell types, such as the colonic epithelium and epithelial cells of the breast and prostate.

The PPAR*γ* gene encodes two main splicing isoforms: PPAR*γ*1 and PPAR*γ*2. PPAR*γ*1 is expressed in many tissues and organs including stomach and small and large intestine, whereas PPAR*γ*2 is expressed specifically in adipocytes [1, 2]. Each PPAR*γ* protein binds as a heterodimer with retinoid X receptor (RXR) to its recognition site called PPRE, with the sequence TGACCTxTGACCT. On binding to DNA, PPAR*γ* recruits transcriptional coactivators such as steroid receptor coactivator 1 (SRC1) and positively regulates the expression of target genes [3, 4]. Though the underlying molecular mechanisms are unclear, PPAR*γ*-mediated transrepression for inflammatory genes has also been described [5].

PPAR*γ* also undergoes several PTMs in response to exogenous signals such as growth factors and adipokines, resulting in modulation of its functions [6]. For example, mitogen-activated protein kinases (MAP kinases) phosphorylate the N-terminal AF-1 domain of PPAR*γ* (serine 84 or 112 in human PPAR*γ*1 or PPAR*γ*2, resp.), and this PTM inhibits ligand-dependent PPAR*γ* transcriptional activity [7–11]. Moreover, PPAR*γ* also undergoes SUMOylation at the C-terminal ligand-binding domain (LBD) in a ligand-dependent manner. SUMOylated PPAR*γ* binds to NCoR corepressor complex and thereby transrepresses inflammatory genes such as iNOS genes in macrophages [12].

In the postgenomic era, it has become obvious that diversity of biological phenomena cannot be explained only by the number of different genes. Protein posttranslational modifications (PTMs) are one of the most efficient biological signals for expanding the genetic code and play key roles in diverse cellular processes such as protein transportation, DNA repair, and gene transcription [13–15]. We have previously developed a comprehensive PTM analysis system for proteins, combining biochemical and proteomic approaches [16]. In this system, we utilized nonrestrictive sequence alignment for PTM analysis and this method made it possible to identify PTMs without prior specification, enabling unbiased analysis of PTMs [17, 18].

Recent findings have revealed that the functions of epigenetic regulators, including PPAR*γ*, are under control of PTMs in response to cellular signaling and nutrients [6, 19]. Here we applied the unrestricted comprehensive analysis to PTMs in PPAR*γ* and identified a subset of PTMs from overexpressed PPAR*γ*2 from 293T cells and endogenous PPAR*γ*1 protein from Caco-2 colon cancer cells.

## 2. Materials and Methods

### 2.1. Plasmids, Antibodies, and Reagents

The expression plasmid for full-length PPAR*γ*2 cDNA was cloned from a cDNA library of human adipocyte [20] and inserted into a pcDNA3 vector (Life Technologies, Carlsbad, CA) with a FLAG tag sequence. Anti-PPAR*γ* antibody (#sc-7273) was purchased from Santa Cruz Biotechnology (Santa Cruz, CA). Troglitazone (Sigma, St. Louis, MO) was dissolved in DMSO.

### 2.2. Cell Culture and Transfection

293T and Caco-2 cells were cultured in Dulbecco's modified Eagle medium (DMEM) plus 10% fetal bovine serum (FBS) and antibiotics. THP-1 cells were cultured in RPMI plus 10% FBS and antibiotics. For transfection of FLAG-PPAR*γ*2 into 293T cells in the 10 cm dishes, we used Lipofectamine 2000 (Life Technologies) according to the manufacturer's instructions and incubated the cells for 24 h.

### 2.3. Purification of the PPAR*γ* Proteins and Immunoprecipitation

Purification of PPAR*γ* protein was performed as previously described [16]. Briefly, we cross-linked 2 *μ*g of antibody with 30 *μ*L of Protein G Dynabeads (Invitrogen) in freshly dissolved 20 mM dimethyl pimelimidate (DMP) in a 0.2 M triethanolamine buffer (pH 8.2). The cross-linking reaction was performed for 1 h at room temperature. The reaction was stopped by replacing the buffer with 50 mM Tris-HCl (pH 7.5).

Cell lysates of 293T transfected with FLAG-PPAR*γ*2 and Caco-2 cell (from a 10 cm dish and 48 dishes, resp.) were prepared with 1% NP-40 buffer (10 mM Tris [pH 7.8], 1 mM EDTA, 150 mM NaCl, and 1% NP-40). Lysates were subjected to immunoprecipitation with Protein G Dynabeads coupled to each antibody for 3 h. The immunoprecipitates were washed by 1% NP-40 buffer and eluted with 0.1 M glycine-HCl buffer (pH 2). The eluates were boiled with Laemmli sample buffer and then subjected to sodium dodecyl sulfate-polyacrylamide gel electrophoresis (SDS-PAGE) and then Colloidal Blue (Life Technologies), silver staining (Silver Quest, Life Technologies), or Western blotting with the indicated antibodies.

### 2.4. Mass Spectrometric Analysis

Mass spectrometric (MS) analysis of PPAR*γ* protein and PTM analysis were performed as previously described [16]. Briefly, PPAR*γ* protein was excised from the gel, reduced with 10 mM dithiothreitol solution in 0.1 M ammonium bicarbonate for 60 min at 56°C, alkylated with a 55 mM solution of iodoacetamide in 0.1 M ammonium bicarbonate in darkness for 45 min at room temperature, and in-gel digested with 25 ng/*μ*L trypsin gold (Promega, Madison, WI) in 50 mM ammonium bicarbonate for 16 h at 37°C. Digested peptides were extracted, replaced with 0.1% formic acid in 2% acetonitrile (ACN), and subjected to analysis by electrospray ionization- (ESI-) MS/MS using an LTQ velos Orbitrap with ETD (Thermo Fisher Scientific). The nano-LC used was a DiNa system (KYA TECH Corporation, Tokyo, Japan) equipped with a C-18 ESI capillary column (100 *μ*m × 150 mm, NIKKYO Technos, Tokyo, Japan). The gradient consisted of (A) 0.1% formic acid in 2% ACN and (B) 0.1% formic acid in 80% ACN: 0–100% B from 0 to 110 min, 100% B from 111 to 115 min, and 0% B from 116 to 120 min. The flow rate was 300 nL/min from 0 to 120 min. MS spectra were recorded from a range of* m*/*z* 350–1500 at 100,000 resolution, followed by data-dependent collision-induced dissociation (CID) MS/MS spectra and electron transfer dissociation (ETD) MS/MS spectra generated from the 20 highest intensity precursor ions. The voltage between the ion spray tip and the transfer tube was set to 1800 V. Peptides with +2 or greater charge were chosen for MS/MS experiments.

### 2.5. Computational Analysis for Protein Identification and PTM Analysis

Protein identification and PTM analysis of PPAR*γ* protein were performed as previously described [16]. Briefly, for protein identification, spectra were processed using Proteome Discoverer ver. 1.3 (Thermo Fisher Scientific) against SEQUEST and subjected to a cutoff of 5% false discovery rate (FDR). The NCBI human protein database was used with a 10 ppm mass accuracy cutoff for parental MS and FT MS/MS and a 0.8 Da cutoff for ion trap MS/MS spectra. Carbamidomethylation (cysteine) was set as a fixed modification and oxidation (methionine) was set as a variable modification.

For PTM identification, spectra were processed using the MODIRO ver. 1.1 (Protagen, Bochum, Germany) software against FASTA format of human PPAR*γ*1 and human PPAR*γ*2 amino acid sequences. Search parameters were set as follows: two maximum missing cleavage sites, a peptide mass tolerance of 15 ppm for peptide tolerance, 1.5 Da for fragment mass tolerance (for ion trap MS/MS), 15 ppm for fragment mass tolerance (for FT MS/MS), and modification 1 of carbamidomethyl (cysteine). In all the identified PTMs, PTMs which might have occurred during sample preparations such as methylation of glutamic acid [21] were excluded from the list.

## 3. Results

### 3.1. Purification of Exogenous PPAR*γ*2 from 293T and Endogenous PPAR*γ*1 from Caco-2 Cells

**Figure 1:**
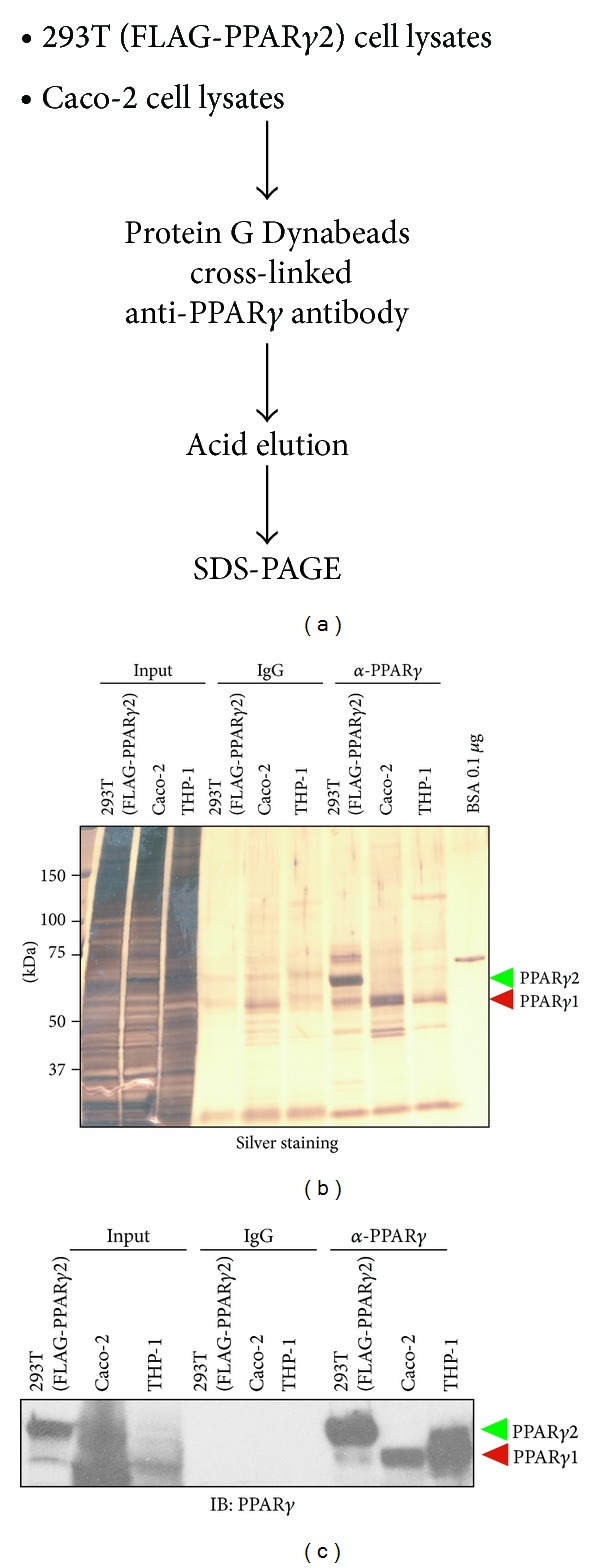
Purification of PPAR*γ* proteins from 293T and Caco-2 cell lysates. (a) Schematic diagram of the purification of the exogenous and endogenous PPAR*γ* proteins. 293T and Caco-2 cell lysates were incubated with anti-PPAR*γ* cross-linked Protein G Dynabeads as described in Section 2. Rabbit IgG cross-linked Protein G Dynabeads were used as a negative control. Bound proteins were eluted with 0.1 M glycine-HCl buffer (pH 2). (b) Isolated exogenous and endogenous PPAR*γ* proteins. Eluted proteins were subjected to SDS-PAGE, followed by silver staining. The molecular masses of PPAR*γ*1 and PPAR*γ*2 are shown in the right side of the gel. (c) Enrichment of PPAR*γ* proteins were also confirmed by Western blotting using anti-PPAR*γ* specific antibodies.

For comprehensive analysis of PTMs in PPAR*γ* protein, we performed the affinity purification of PPAR*γ* proteins from 293T cells and Caco-2 colon cancer cells, which were expressing exogenous FLAG-tagged PPAR*γ*2 and endogenous PPAR*γ*1 protein, respectively, using the scheme shown in Figure 1(a). 1% NP-40 soluble fraction of cell lysates prepared from 293T and Caco-2 cells without PPAR*γ* ligand was prepared and incubated with Protein G Dynabeads cross-linked with anti-PPAR*γ* antibodies. Purified protein-antibody complexes were washed with lysis buffer and then eluted from the antibodies by adding acidic glycine-HCl buffer; eluates were then subjected to SDS-PAGE and visualized by silver staining. As shown in Figure 1(b), we successfully detected exogenous FLAG-PPAR*γ*2 in the anti-PPAR*γ* affinity purified eluates at the expected molecular size. We also detected endogenous PPAR*γ*1 proteins in the anti-PPAR*γ* affinity purified eluates from Caco-2. THP-1 human monocyte cells were used as indicators of PPAR*γ*1 molecular size [22]. Enrichment of FLAG-PPAR*γ*2 and endogenous PPAR*γ*1 proteins was also confirmed by Western blotting using anti-PPAR*γ* specific antibodies (Figure 1(c)).

### 3.2. Identification of PPAR*γ* Proteins Using SEQUEST Algorithm

**Figure 2:**
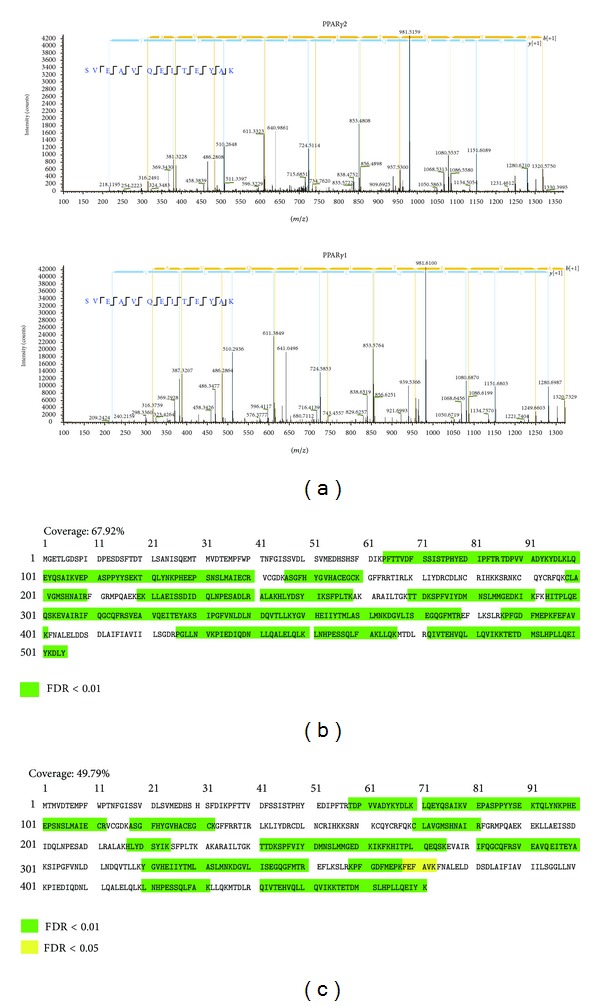
Identification of PPAR*γ* proteins using the SEQUEST algorithm. (a) Representative MS/MS spectra of PPAR*γ*2 proteins of the peptide [317-SVEAVQEITEYAK-329] and PPAR*γ*1 proteins of the peptide [289-SVEAVQEITEYAK-301] assigned by SEQUEST are shown. (b) Amino acid sequence coverage of identified exogenous PPAR*γ*2 proteins. The identified amino acid sequence is indicated. (c) Amino acid sequence coverage of identified endogenous PPAR*γ*1 proteins. The identified amino acid sequence is indicated.

The purified FLAG-PPAR*γ*2 from 293T cells and PPAR*γ*1 protein from Caco-2 cells were cut from the colloidal blue-stained gel, in-gel digested with trypsin, and subjected to analysis by LC-MS/MS using a combination of collision-induced dissociation (CID) and electron transfer dissociation (ETD) activation. Precursor MS spectra were detected by Orbitrap mass analyzers (a Fourier transform (FT) mass analyzer that can measure peptide* m*/*z* with high accuracy) (ΔMS < 5 ppm), and MS/MS spectra were detected using the ion trap analyzer (ΔMS < 0.5 Da). MS spectral data were first analyzed using the SEQUEST algorithm in Proteome Discoverer ver. 1.3 and the PPAR*γ* protein was identified with high accurate molecular weight (sequence coverage 67.92%, FDR < 5% for PPAR*γ*2 from 293T cells, coverage 49.79%, FDR < 5% for PPAR*γ*1 from Caco-2 cells), suggesting successful protein purification and mass measurement for the PPAR*γ* proteins (Figures 2(a), 2(b) and 2(c)).

### 3.3. Nonrestrictive PTM Analysis for Purified PPAR*γ* Proteins Using the MODIRO Algorithm

**Figure 3:**
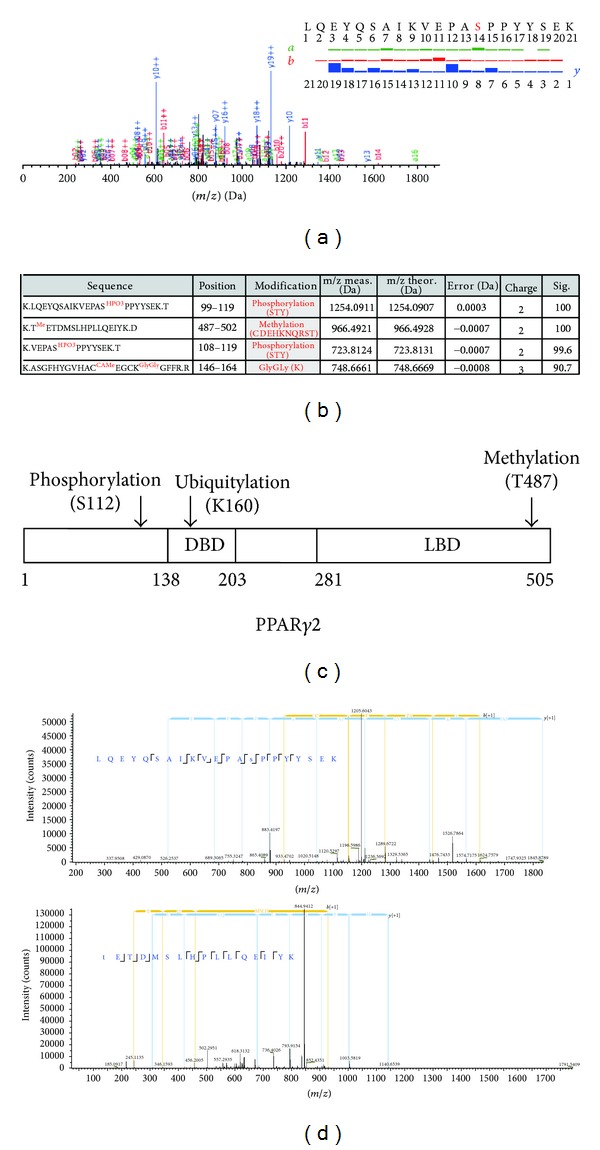
Identified PTMs in PPAR*γ*2 proteins from 293T cells. (a) Representative MS/MS spectra of PPAR*γ*2 proteins of the peptide [99-LQEYQSAIKVEPAS^HPO3^PPYYSEK-119] assigned by MODIRO are shown. (b) List of identified PTMs in exogenous PPAR*γ*2 proteins using ion trap MS/MS. Amino acid sequences, position of amino acids, identified PTMs theoretical mass, measured mass, error between measured and theoretical masses in Daltons, and significance score are listed. (c) Diagram for summary of identified PTMs in exogenous PPAR*γ*2 proteins. (d) Identified PTMs in exogenous PPAR*γ*2 proteins using FT MS/MS. Each responsible spectrum for S112 (top) and T487 (bottom) is shown.

Next, the spectral data were searched using the MODIRO algorithm, which enables unrestricted identification of all possible PTMs in targeted proteins. As a result, various PTMs were identified in peptides derived from exogenous PPAR*γ*2 protein from 293T cells (Figures 3(a) and 3(b)), and identified PTMs with significance score >90 were presented in Figures 3(b) and 3(c). At the top of the list, phosphorylation of serine 112 in PPAR*γ*2 was identified with significance score = 100. This phosphorylation site is one of the well-characterized PTM sites in PPAR*γ*2 protein, catalyzed by MAPKs and inhibiting the ligand-dependent transcription function of PPAR*γ* [7–11]. This result confirms that our experimental approach detects the PTMs of PPAR*γ* protein correctly. Furthermore, this PTMs list also included some PTMs such as methylation of 487 threonine residue and ubiquitination of lysine 160, which were novel PTMs for PPAR*γ* proteins.

To further confirm these identified PTMs, we analyzed the peptides by LC-MS/MS again. In this measurement, to obtain more rigorous MS/MS data and thereby more reliable PTM search results, we utilized the FT analyzer for both parental MS and MS/MS spectra. As shown in Figure 3(d), two of the three PTMs including S112 phosphorylation and T487 methylation were identified again with SEQUEST algorithm, thus confirming the initial result.

**Figure 4:**
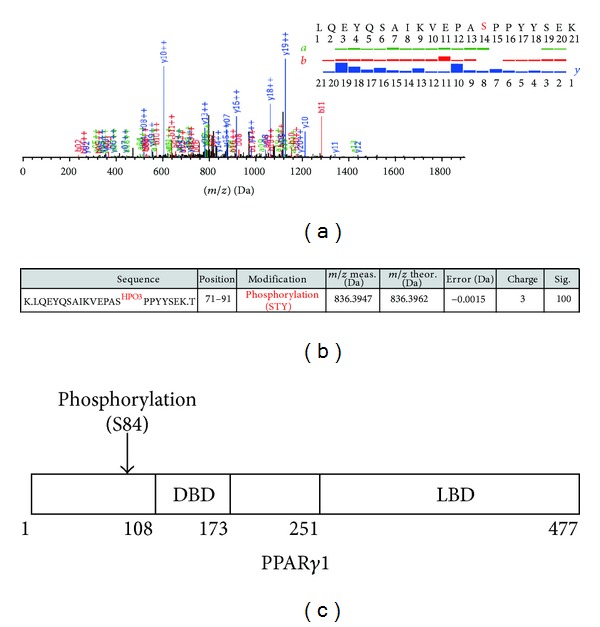
Identified PTMs in PPAR*γ*1 proteins from Caco-2 cells. (a) Representative MS/MS spectra of PPAR*γ*1 proteins of the peptide harboring [80-VEPAS^HPO3^PPYYSEK-91] assigned by MODIRO are shown. (b) List of identified PTMs in endogenous PPAR*γ*1 proteins using ion trap MS/MS. (c) Diagram for summary of identified PTM in endogenous PPAR*γ*1 proteins.

Next, PPAR*γ*1 proteins from Caco-2 cells were analyzed in the same way. As shown in Figures 4(a), 4(b) and 4(c), phosphorylation of serine 84 was identified with significance score = 100. This serine residue corresponds to serine 112 of PPAR*γ*2. We further tested PPAR*γ*1 proteins from 10 *μ*M Troglitazone-treated Caco-2 cells (24 h). However, no other PTMs besides S84 phosphorylation were identified from this analysis (data not shown).

## 4. Discussion

Here, we described the first report of comprehensive PTM analysis of PPAR*γ* protein using mass spectrometry and succeeded in identifying some previously known and unknown PTMs including serine phosphorylation and threonine methylation.

Among the identified PTMs, phosphorylation of S112 and S84 in PPAR*γ*2 and PPAR*γ*1, respectively, was the one of the well-known PTMs in PPAR*γ* proteins. It is reported that phosphorylation at S112 in PPAR*γ*2 was catalyzed by a mitogen-activated protein (MAP) kinase and was involved in repression of transcriptional activity in adipocyte [7]. However, whether this modification exists in the PPAR*γ*1 protein in colon cancer cells was elusive. Thus, in this study we could confirm the existence of S84 phosphorylation in PPAR*γ*1 in colon cancer cells. Although the role of S84 phosphorylation in colon cancer is unclear, the phosphorylation mimic mutant of PPAR*γ*1 (S84D) slightly derepressed PPAR*γ*-mediated transrepression of *β*-catenin using the TOPflash reporter system [23], and further characterization with regard to the effect in transcriptional function is needed (Shogo Katsura, unpublished result).

In the present study, we could also identify the K160 ubiquitination and T487 methylation of PPAR*γ* proteins, although there are few reports about these modifications. Protein ubiquitination can be targeted to a number of biological pathways such as proteasomal degradation and signal transduction depending on the numbers and linkage types of the conjugated ubiquitins [24]. Because the number and linkage types of the conjugated ubiquitins were not clear from present experiment, further detailed analyses are required to characterize the function of K160 ubiquitination in PPAR*γ*2 protein. As for threonine methylation, it is still unclear whether this modification occurs in the sample preparation prior to MS analysis or is posttranslational [14]. We avoided methanol in each step of our experimental procedure so that proteins were not nonenzymatically methylated, although still we should be careful about this modification. However, this is a novel modification of PPAR*γ* protein and thus our comprehensive analysis of PTMs can be applied to find previously unidentified PTMs in PPAR*γ* proteins and other proteins including nuclear receptors.

Along with the identified phosphorylation, ubiquitination, and methylation, PPAR*γ* protein is known to also undergo several PTMs, such as phosphorylation at other regions [25, 26], SUMOylations [12],* O*-GlcNAcylations [27], and ubiquitination [28, 29], although these PTMs were not identified in this study. Generally, as mass spectrometric analysis can detect relatively highly concentrated or efficiently ionized peptides [15, 17], it is possible to speculate that there are many more PTMs than what have been identified in PPAR*γ* proteins. Because purified PPAR*γ* proteins might be mixtures of variously modified states, we presume that, with further purification of the eluted proteins such as by isoelectric fractionation of purified PPAR*γ* proteins or enrichment of specific modifications with PTM-recognizing antibodies, we could extend the variations of identified peptides and thus detect greater numbers of PTMs in PPAR*γ* proteins. Those PTMs might be new candidates for drug targets to control PPAR*γ* activities.

## 5. Conclusions

We have identified several modifications including phosphorylation of serines 112 and 81 in PPAR*γ*2 and PPAR*γ*1, respectively, and threonine methylation in PPAR*γ*2 using our previously established PTM analysis system. This method can be applied to find previously unidentified PTMs in PPAR*γ* proteins and other proteins including nuclear receptors.
